# Assessment of Corneal Quality by Eye Banks

**Published:** 2011-01

**Authors:** W John Armitage

**Affiliations:** CTS Bristol Eye Bank, Bristol Eye Hospital, University of Bristol, Bristol, UK

Assurance of the quality and safety of corneas meant for transplantation is a key responsibility of eye banks. While donor testing and medical assessment mitigate the risk of disease transmission from donor to recipient, the quality of corneas is crucial to successful transplantation in terms of both graft survival and visual rehabilitation. With the development and increasing application of lamellar techniques for selective replacement of dysfunctional layers of the cornea,^1^ different aspects of corneal quality need to be prioritized by eye banks and corneal transplant surgeons, depending on the intended surgical procedure. Penetrating keratoplasty and endothelial keratoplasty still account for the majority of corneal transplants and, for these procedures, endothelial function is essential for restoring corneal transparency and vision. The principal measure used by eye banks for assessing corneal quality for these procedures is endothelial cell density. This is entirely reasonable, given the accelerated decline in endothelial cell density after transplantation.^2^ Late endothelial failure remains a major risk in corneal transplants that have not succumbed to failure from allograft rejection, disease recurrence, or other specific causes of graft failure.^3^ However, it is also important to take into account a range of quality criteria and to be able to explain and interpret the significance of seemingly abnormal features that become apparent with increasing post-mortem time, before eye retrieval and during corneal storage.

Although there is currently little rationale for setting minimum donor endothelial cell densities,^2^ which typically vary from 2000 to 2500 cells/mm^2^, it is self-evident that endothelial cell density will have an impact on long-term graft survival. It is more difficult to assess the influence of, for example, pleomorphism and polymegathism or qualitative measures of endothelial quality, such as the presence of Descemet’s folds, on graft outcome. The significance of some features is, however, amenable to laboratory investigation. One such example is reported in this issue, in a paper by Mozhgan Rezaei Kanavi and colleagues^4^ on corneal endothelial vacuolation.

The authors performed a useful analysis by light and electron microscopy of dark patches observed at the level of the corneal endothelium by slit lamp biomicroscopy and specular microscopy. While this apparent vacuolation is a criterion applied by eye banks in the overall assessment of quality, its significance and relevance to endothelial health is revealed by this study. The investigators point out that these patches could be caused by precipitates or even red blood cells; moreover, if the changes were to be reversible, their relevance to quality would be called into question. However, it is clear from the authors’ careful observations that these patches are indeed a result of cellular changes, and they are confirmed to be cytoplasmic vacuoles causing membrane bleb formation. These findings strongly suggest that these features are associated with poorer endothelial quality, especially if as suggested, they are indicative of apoptotic changes. It would be interesting to further test this supposition with immunohistochemical markers for apoptosis. In summary, not only has the nature of these dark patches been revealed, but there is now justification for associating these patches with deleterious changes in the endothelium that could compromise a successful graft outcome.

These observations are certainly of value to eye banks, since they contribute to our overall understanding of the quality assessment criteria for corneas designated for transplantation.
